# Do CAD-CAM fibre posts exhibit higher bond strength and fracture resistance than other types of posts? systematic review with network meta-analysis of in-vitro studies

**DOI:** 10.1038/s41405-025-00315-x

**Published:** 2025-03-17

**Authors:** Mohammed Maher Ghanem, Xin Yi Leong, Sajesh K. Veettil, Anas Al Jada, Musab Saeed, Rohit Kunnath Menon

**Affiliations:** 1 Master’s student, School of Dentistry, Ajman University, Ajman, United Arab Emirates; 2 Graduate, College of Dentistry, International Medical University, Kuala Lumpur, Wilayah Persekutuan Malaysia; 3 School of Pharmacy, Department of Pharmacy Practice, College of Pharmacy, International Medical University, Kuala Lumpur, Wilayah Persekutuan Malaysia; 4 Clinical Sciences Department, College of Dentistry, Ajman University, Ajman, United Arab Emirates; 5 Clinical sciences department, College of Dentistry, Ajman University, Ajman, United Arab Emirates

**Keywords:** Dentistry, Prosthetic dentistry

## Abstract

**Introduction:**

Pre-fabricated fibre posts facilitate post-endodontic restoration of endodontically treated teeth with insufficient coronal tooth structure. Recent advancements in digital dentistry have led to the introduction of custom-made fibre posts fabricated with computer-aided design-computer-aided manufactured (CAD-CAM) technology. However, evidence on the comparative performance of the different post-types is lacking. This systematic review with network meta-analysis aimed to analyse the current evidence on in-vitro studies comparing bond strength, catastrophic failures, fracture resistance, and cement film thickness between CAD-CAM fibre posts and other post types, including pre-fabricated fibre and custom-made cast-metal posts.

**Materials and methods:**

A systematic search was conducted in PubMed, Scopus, and the Cochrane Central Register of Controlled Studies for in-vitro studies from inception until December 2023 (PROSPERO: CRD42024501614). Network meta-analysis and pairwise meta-analysis were performed. The ranking was performed using the surface area under the cumulative ranking (SUCRA) guidelines.

**Results:**

Seven hundred forty-one articles were identified, of which 80 duplicates were removed, and 585 were excluded by screening the titles and abstracts. A total of 76 articles were assessed by full-text reading, and 16 were included in the quantitative synthesis. CAD-CAM fibre posts (SMD = 1.09 [95% CI: 0.01, 2.17]) *P* = 0.04 demonstrated higher bond strength when compared to pre-fabricated posts. CAD-CAM fibre posts (RR = 0.39 [95% CI: 0.23, 0.69]) *P* < 0.05 demonstrated a lower risk for catastrophic failures when compared to pre-fabricated fibre posts.

**Conclusions:**

In-vitro studies demonstrated that CAD-CAM fibre posts demonstrated higher bond strength, lower catastrophic failure rates, and similar fracture resistance compared to pre-fabricated and cast metal posts. The data on bond strength and catastrophic failures of CAD-CAM fibre posts must be validated clinically by high-quality, randomised, controlled clinical trials.

## Introduction

Root canal-treated teeth with insufficient coronal tooth structure require a custom or pre-fabricated post system to retain the core material before fabrication of an indirect restoration. Various post systems have been used previously to gain intracanal support to support an extrinsic core material. Customised fabrication of posts involves making an impression of the root canal configuration, followed by the fabrication of a post which closely adheres to the root canal morphology. Custom cast posts show better resistance to torsional stress [1, 2] in the teeth with flared and multiple roots [3, 4]. Custom posts can be fabricated by employing a direct or indirect technique from metallic or non-metallic materials [5]. Metal custom posts have previously reported acceptable success rates and mechanical qualities [6]. Superior aesthetics and fracture resistance have been attributed to ceramic posts [7]. However, posts made of zirconia have exhibited reduced attachment strength and an increased risk for root fractures [8, 9]. Custom posts can be fabricated conventionally or by a computer-aided design-computer-aided manufacturing (CAD-CAM) protocol [10–13]. Pre-fabricated posts can be fabricated from different metallic and non-metallic materials. Titanium and stainless steel posts exhibit diminished radiographic visibility and corrosion, respectively [14]. Ceramic posts demonstrate higher strength and aesthetics but diminished resin bonding [15–22]. Fibre posts report a much lower incidence of root fractures [23, 24], acceptable aesthetics and retrievability [25]. A recent systematic review provides robust evidence on pre-fabricated fibre posts’ ability to reduce the risk of secondary caries, debonding, and tooth fracture [26]. CAD-CAM-fabricated glass fibre posts have been reported to exhibit enhanced fracture resistance, bond strength, retention and aesthetics compared to pre-fabricated glass fibre posts [27–31]. Comparative studies between CAD-CAM fibre posts and other types of posts are required to generate evidence regarding the acceptability of CAD-CAM posts. Network meta-analysis (NMA) enables investigators to combine direct and indirect evidence to establish comparative efficacy across a network of studies. The purpose of this NMA was to compare the bond strength, fracture resistance, catastrophic failure, and cement film thickness of CAD-CAM custom fibre posts and other types of posts from in vitro studies.

## Materials and methods

The protocol for the systematic review was registered with the International Prospective Register of Systematic Reviews (PROSPERO: (CRD42024501614)) and reported according to the Preferred Reporting Items for Systematic Reviews and Meta-Analysis (PRISMA) extension statement of NMA [32]. The study population included teeth that are endodontically treated, followed by post and core. Interventions were CAD-CAM fibre posts. Comparators were pre-fabricated fibre posts or other types of posts. The outcomes evaluated were fracture resistance, bond strength, catastrophic failures, and cement film thickness. Screening of titles and abstracts, full-text reading, and data extraction were performed independently and in duplicates by two reviewers (MMG, XYL). The risk of bias within each study was independently assessed by two reviewers (MMG, XYL) using RoBDEMAT (RoB 2.0) [33]. Disagreements and discrepancies were resolved by discussion with a third reviewer (R.K.M). Standardised mean differences (SMD) in millimetres (mm) and 95% confidence intervals were used as summary statistics for continuous outcomes. For direct comparisons, a standard pairwise meta-analysis was performed by using a random-effects (DerSimonian and Laird) model [32, 34]. If a direct comparison was based on two or more studies, heterogeneity among trials was assessed by considering the I^2^ statistics [32, 35]. To synthesise the available evidence by combining direct and indirect evidence from different studies, a random-effects NMA was applied [36–38]. The probability of each post type being the best was estimated by constructing nanograms and their surface area under the cumulative ranking (SUCRA) [38, 39]. A comparison-adjusted funnel plot was used to examine the publication bias. A statistical software programme (Stata version 15.0; (StataCorp)) was used for statistical analysis and graph generation [40].

## Results

**Fig. 1:**
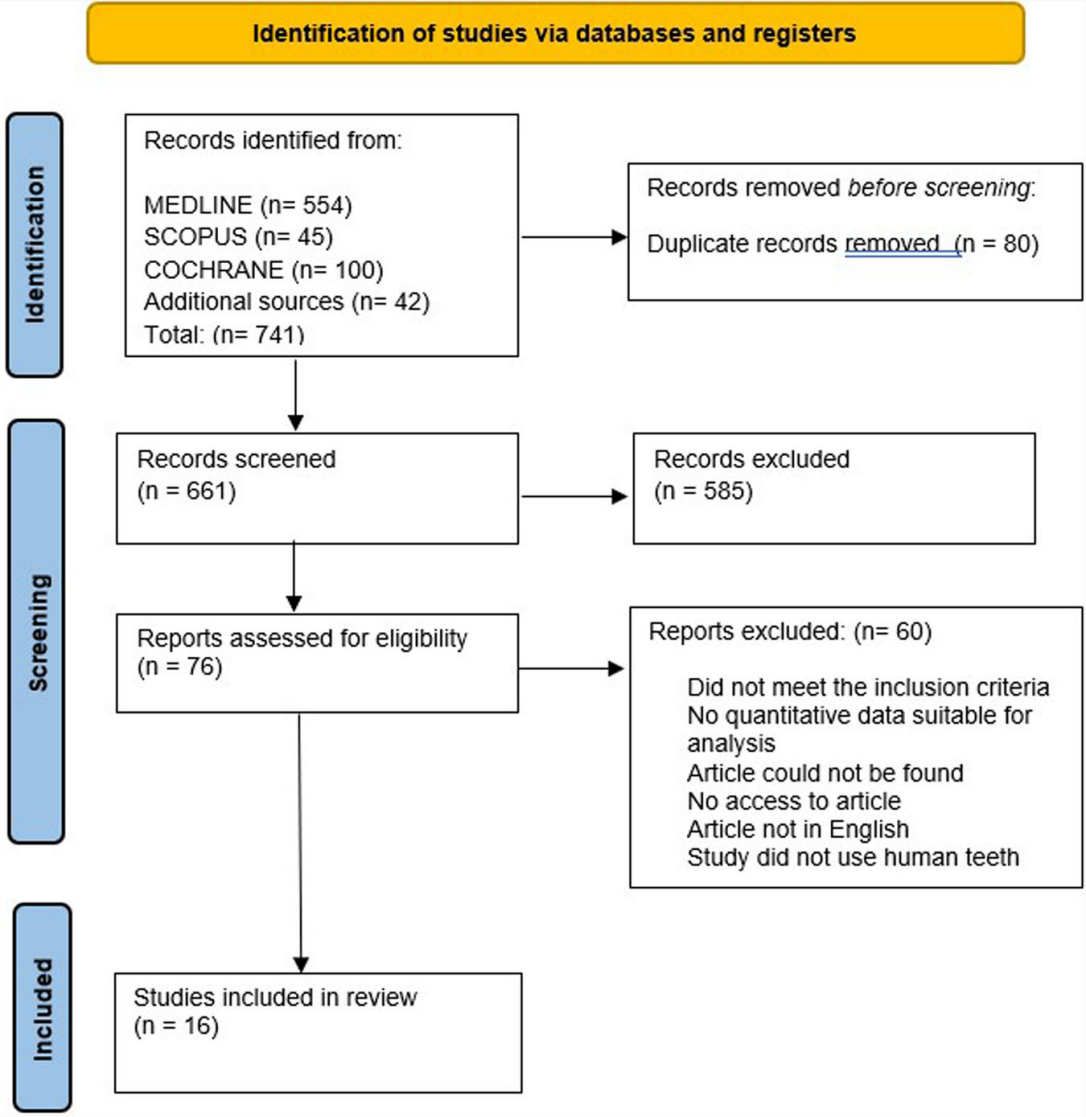
PRISMA Flowchart describing the search strategy and identification of articles. PRISMA-Preferred Reporting Items for Systematic Reviews and Meta-Analysis, n-number.

**Fig. 2:**
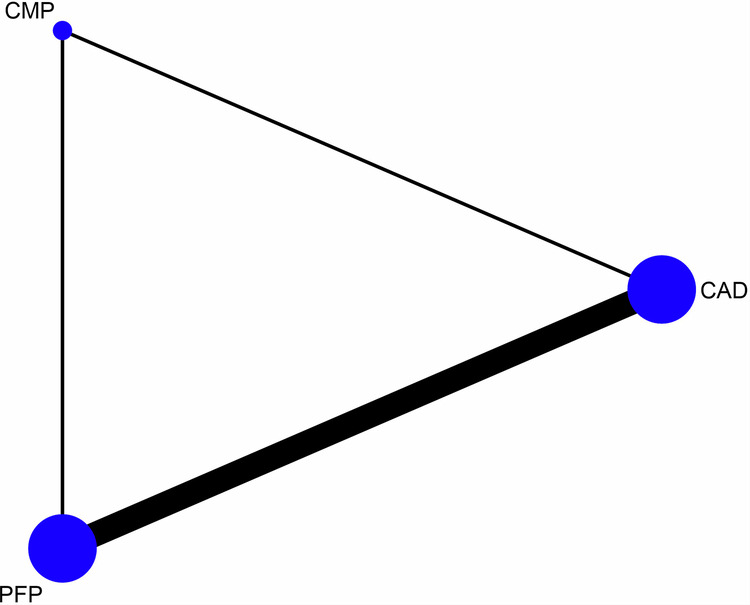
Network plot depicting the network meta-analysis results for bond strength. CAD-CAD-CAM fibre posts PFP-Pre-fabricated fibre posts, CMP cast metal posts.

**Fig. 3:**
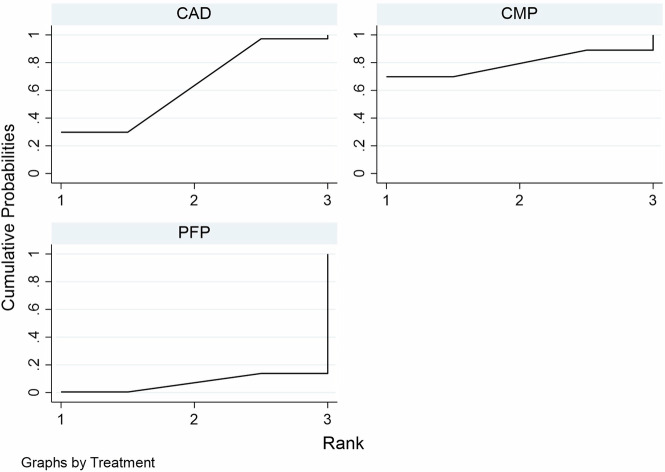
SUCRA ranking curves for bond strength. CAD-CAD-CAM fibre posts PFP-Pre-fabricated fibre posts, CMP cast metal posts.

**Fig. 4:**
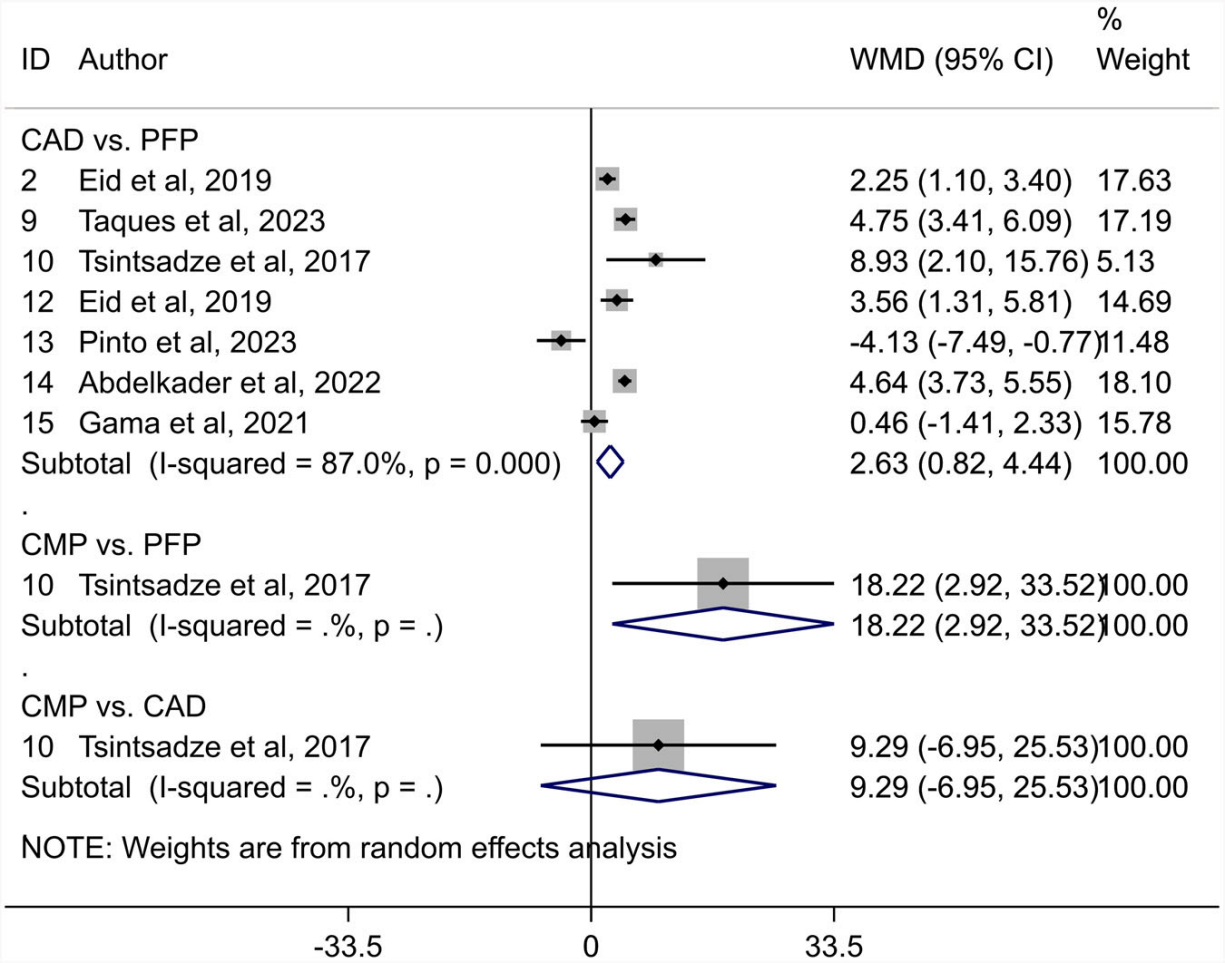
Forest plot depicting the pairwise meta-analysis results for bond strength. CAD-CAD-CAM fibre posts PFP-Pre-fabricated fibre posts, CMP cast metal posts.

**Fig. 5:**
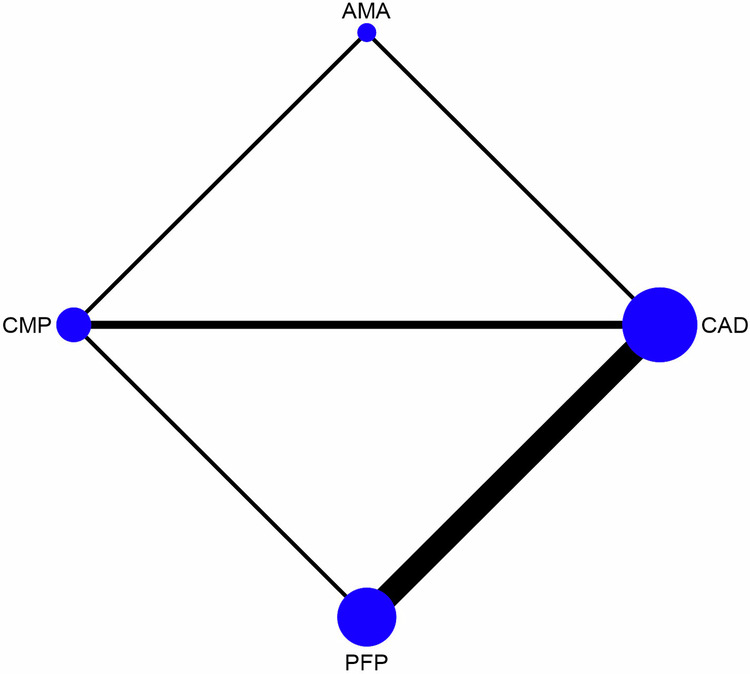
Network plot depicting the network meta-analysis results for catastrophic failures. CAD-CAD-CAM fibre posts PFP-Pre-fabricated fibre posts, CMP cast metal posts, AMAAmalgam cores.

**Fig. 6:**
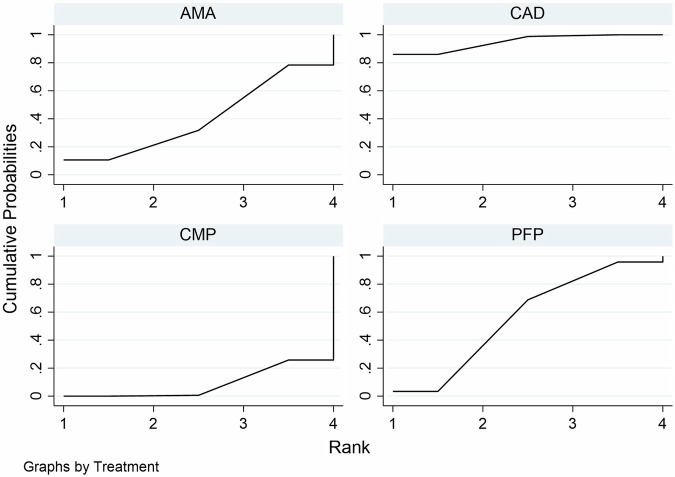
SUCRA ranking curves for catastrophic failures. CAD-CAD-CAM fibre posts PFP-Pre-fabricated fibre posts, CMP Cast metal posts, AMA Amalgam cores.

**Fig. 7:**
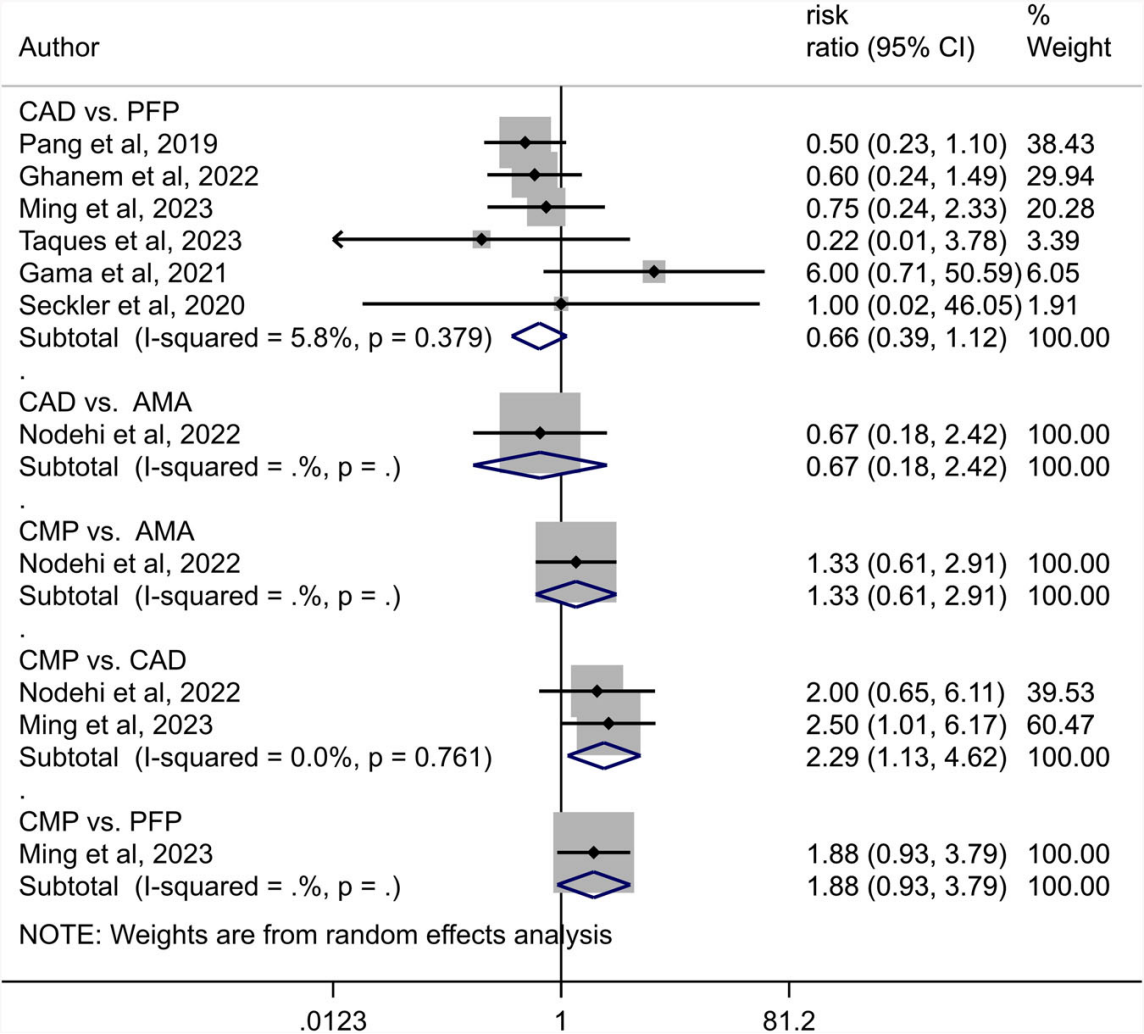
Forest plot depicting the pairwise meta-analysis results for catastrophic failures. CAD-CAD-CAM fibre posts PFP-Pre-fabricated fibre posts, CMP cast metal posts, AMA amalgam cores.

A total of 741 articles were initially identified, of which 80 duplicates were removed, and 585 were excluded by screening the titles and abstracts. A total of 76 articles were assessed by full-text reading. Sixteen articles were selected for the quantitative analysis [27, 41–55]. The preferred reporting items for systematic reviews and meta-analyses (PRISMA) flow diagram (Fig. 1. Table 1 shows the characteristics of the included studies. The quality assessment of each study using the RoBDEMAT assessment tool is provided in Table 2. CAD-CAM fibre posts (CAD), pre-fabricated fibre posts (PFP), cast metal posts (CMP), and amalgam cores (AMA) were compared. For bond strength, seven in vitro studies comparing three interventions were included in the NMA (Fig. 2). CAD (SMD = 1.09 [95% CI: 0.01, 2.17]) *P* = 0.04 demonstrated higher bond strength when compared to PFP. Supplementary Table 1 summarises the SMD and the ranking of the interventions, while Fig. 3 shows the SUCRA ranking curves for each intervention in the network. CMP ranked the highest, followed by CAD and PFP, respectively. The results of the pairwise meta-analysis are depicted in the forest plot in Fig. 4. CAD (SMD = 2.63 [95% CI: 0.82, 4.44]) *P* < 0.04 demonstrated higher bond strength when compared to PFP. The network and pairwise estimates for all the interventions are summarised in Supplementary Fig. 1. Based on the comparison-adjusted forest plots, publication bias could be detected (Supplementary Fig. 2). For catastrophic failure, seven in vitro studies comparing four interventions were included in the NMA (Fig. 5). None of the interventions demonstrated a significant difference in catastrophic shortcomings compared to PFP. Supplementary Table 2 summarises the SMD and the ranking of the interventions, while Fig. 6 shows the SUCRA ranking curves for each intervention in the network. The results of the pairwise meta-analysis are depicted in the forest plot, Fig. 7. CMP (RR = 2.29 [95% CI: 1.13, 4.62]) *P* < 0.05 demonstrated a higher risk for catastrophic failure when compared to CAD. The network and pairwise estimates for all the interventions are summarised in Supplementary Fig. 3. CAD (RR = 0.39 [95% CI: 0.23, 0.69]) *P* < 0.05 demonstrated a lower risk for catastrophic failure when compared to PFP. Based on the comparison-adjusted forest plots, publication bias could be detected (Supplementary Fig. 4). For fracture resistance, seven in vitro studies comparing two interventions were included in the pairwise meta-analysis as depicted in the forest plot, Supplementary Fig. 5. None of the comparisons were statistically significant. Inconsistency was identified in the NMA for cement film thickness, and no significant findings were observed in the pairwise analysis. The network plot (Supplementary Fig. 6), SUCRA ranking curves (Supplementary Fig. 7) and the forest plot (Supplementary Fig. 8) are provided.

**Table 1:** Characteristic table describing the characteristics of the included studies.

Author/Year	Group/Sample size	Cement	Outcomes measured	Test used
Ming et al. [49]	Group1: Traditional casting titanium (Ti) post-cores (TC group). (*n* = 8) Group2: Ti post-cores fabricated with selective laser melting (SLM group). (*n* = 8) Group3: CAD/CAM glass fibre post-cores of the split type (CCS group).(*n* = 8) Group4: Pre-fabricated glass fibre posts and composite resin cores (PF group).(*n* = 8)	Self-adhesive resin cement (RelyX Unicem, 3 M ESPE)	Internal adaptation. Fracture resistance Fracture pattern.	Microcomputed tomography. Fracture resistance test
Pinto et al. [52]	Group 1: Pre-fabricated GFPs (glass fibre posts). (*n* = 15) Group 2: Milled and customized CAD-CAM posts. (*n* = 15)	Panavia 2.0 (Kuraray)	Bond Strength Failure mode.	Push-out test. Stereomicroscope at 40x magnification
Saisho et al. [47]	Group1:Polyetheretherketone (PK; Ceramill PEEK).(*n* = 20) Group2: Nanohybrid composite resin (BB; Brava Block).(*n* = 20) Group3: Polymer-infiltrated ceramic (EN; VITA Enamic).(*n* = 20) Group4: Fibre-reinforced epoxy resin (GF; Fibre Cad Post & Core).(*n* = 20)	Dual-polymerizing composite resin luting agent (Allcem Core; FGM)	Fracture strength. Pull-out bond strength (POBS)	Fracture strength testing. POBS testing.
Taques et al. [51]	Group 1: Pre-fabricated glass fibre post [PFP]. (*n* = 16) Group 2: Direct anatomical glass fibre post [AFP]. (*n* = 16) Group 3: CAD/CAM milled glass fibre post [MFP]. (*n* = 16)	RelyX Ultimate (3 M ESPE)	Bond Strength. Fracture Strength. Failure mode.	Push-out test. Compression test.
Abdelkader et al. [53]	Group 1: Post space prepared with the #0.8 drill, receive a #0.8 pre-fabricated glass fibre post. (*n* = 10) Group 2: Post space prepared to an oversized root canal, receive a #0.8 pre-fabricated glass fibre post. (*n* = 10) Group 3: CAD-CAM glass fibre post. (*n* = 10)	G-CEM LinkAceTM, (GC Corp)	Bond Strength (MPa).	Push-out test.
Ghanem et al. [50]	Group1:MPP: restored with custom-milled posts and cores fabricated from polyetherketoneketone (PEKK).(*n* = 10) Group2:MFP: restored with custom-milled posts and cores fabricated from fibre-reinforced resin. (*n* = 10) Group3:PFP: restored with pre-fabricated fibre-reinforced resin posts.(*n* = 10)	Self-adhesive resin cement (TheraCem, Bisco, USA)	Fracture resistance. Failure modes.	Fracture resistance test
Nodehi et al. [48]	Group 1: Restored with amalgam (control).(*n* = 5) Group 2: Post and core by cobalt-chromium casting (Co-Cr).(*n* = 5) Group 3: Post and core by non precious gold (NPG) casting.(*n* = 5) Group 4: Post and core by CAD-CAM milling.(*n* = 5)	Glass ionomer cement (GCCo,Tokyo,Japan)	Fracture resistance (N) Mode of failure	Fracture test
Gama et al. [54]	Group 1: (GPF) received pre-fabricated posts. (*n* = 10) Group 2: (GREL) received relined glass fibre posts. (*n* = 10) Group 3: (GMILLED) received CAD/CAM milled glass fibre posts. (*n* = 10)	RelyX U200 (3 M ESPE)	Bond Strength. Fracture Strength Failure mode.	Push-out bond strength test. X-ray microcomputed tomography analysis.
Seckler et al. [55]	Group 1: (GFP): Glass fibre post and core customized with composite resin. (*n* = 10) Group 2: (CPC): Cast metal. (*n* = 10) Group 3: CAD/CAM-fabricated glass fibre post and core. (*n* = 10)	RelyX U200 (3 M ESPE)	Fracture resistance. Failure mode.	Compression test
Eid et al. [43]	Group 1: BLC (Trilor, Bioloren).(*n* = 20) Group 2:AMC: (Ambarino, Creamed).(*n* = 20) Group 3: BLP:(Bioloren; Filtek Bulk Fill Posterior, 3 M).(*n* = 20) Group 4:RXP:(RelyX fibre post, 3 M; Filtek Bulk Fill Posterior).(*n* = 20)	(RelyX U200 Automix, 3 M ESPE, St Paul, MN)	Bond strength.	Micro-push-out test
Eid et al. [45]	Group1:(RXP) :(Rely X, 3M-ESPE) with (Filtek Bulk Fill Posterior, 3M-ESPE).(*n* = 10) Group2:(BLC) :(Trilor, Bioloren).(*n* = 10) Group3:(AMC) :(Ambarino, Creamed).(*n* = 10)	(Rely X U200, 3 M ESPE)	Fracture resistance	Fracture test
Eid et al. [46]	Group 1 (CP): CAD/CAM fibre-reinforced resin posts. (*n* = 10) Group 2 (CPL): CAD/CAM fibre-reinforced resin posts lubricated with Vaseline. (*n* = 10) Group 3 (RXP): pre-fabricated glass-fibre posts. (*n* = 10)	RelyX U200, 3 M ESPE (Self adhesive cement)	Bond strength. Failure mode.	Push out test. Stereo microscope.
Pang et al. [41]	Group1: CAD/ CAM integrated glass fibre post-and-core.(*n* = 10) Group2: Pre-fabricated glass fibre posts, (Matchpost® RADIOPAQUE, RTD Dental, St E grève, France) and composite resin cores (Filtek™ Z350XT, 3 M ESPE, St. Paul, MN, USA).*(n* = 10) Group3:Pre-fabricated cast gold alloy post-and-core (ARGEDENT Y73, ARGEN, San Diego, CA, USA).(*n* = 10)	(RelyX™ Unicem 2, 3 M, St. Paul, MN, USA)	Fracture resistance.	Fatigue test
da Costa et al. [27]	Group 1 (PPc): Pre-fabricated GFP with crown.(*n* = 10) Grupo 2 (PPn): Pre-fabricated GFP without crown.(*n* = 10) Grupo 3 (CPc): CAD/CAM GFP with crown Grupo 4 (CPn): CAD/CAM GFP without crown.(*n* = 10) Grupo 4 (CPn): CAD/CAM GFP.(*n* = 10)	Allcem Core, FGM	Fracture resistance. Failure mode. Cement layer thickness(μm)	Fatigue testing Observational -micro-CT.
Passos et al. [44]	Group 1 (VE): CAD/CAM glass-fibre posts without ferrule. (*n* = 10) Group 2 (VEF): CAD/CAM glass-fibre posts with ferrule. (*n* = 10) Group 3 (WP): Pre-fabricated glass-fibre posts without ferrule. (*n* = 10) Group 4 (WPF): Pre-fabricated glass-fibre posts with ferrule. (*n* = 10)	RelyX U200, 3 M ESPE (Self-adhesive cement)	Fracture resistance. Failure mode.	Fracture testing. Optical microscope.
Tsintsadze et al. [42]	Group 1: Pre-fabricated GFP: glass fibre posts.(*n* = 10) Group 2: CP: Cast posts.(*n* = 10) Group 3: CAD/ CAM GFP: glass fibre posts.(*n* = 10) (*n* = 6: (push-out) (*n* = 4 (nanofiltration)	Gradia Core (GC) Dual cure cement	-Bond strength. (MPa). -Cement layer thickness. (μm) -Nanoleakage	Push out test Scanning electron microscopy. Interfacial nanoleakage in AgNO3

**Table 2:** Risk of bias analysis performed by ROBDemat.

Author	Year	Domain 1: Bias in planning and allocation			Domain 2: Bias in sample/specimen preparation		Domain 3: Bias in outcome assessment		Domain 4: Bias in data treatment and outcome reporting	
		1.1: Control group	1.2: Randomization of samples	1.3: Sample size rationale and reporting	2.1: Standardization of samples and materials	2.2: Identical experimental conditions across groups	3.1: Adequate and standardized testing procedures and outcomes	3.2: Blinding of the test operator	4.1: Statistical analysis	4.2: Reporting study outcomes
Ming et al.	2023	SR	IR	NR	SR	SR	SR	NR	SR	SR
Pinto et al.	2023	SR	IR	SR	SR	SR	SR	NR	SR	SR
Saisho et al.	2023	SR	IR	NR	SR	SR	SR	NR	SR	SR
Taques et al.	2023	SR	IR	NR	SR	SR	SR	NR	SR	SR
Abdelkader et al.	2022	SR	NR	NR	IR	SR	SR	NR	SR	SR
Ghanem et al.	2022	SR	IR	NR	SR	SR	SR	NR	SR	SR
Nodehi et al.	2022	SR	IR	NR	SR	SR	SR	NR	SR	SR
Gama et al.	2021	SR	NR	NR	IR	SR	SR	NR	SR	SR
Seckler et al.	2020	SR	SR	SR	IR	SR	SR	NR	SR	SR
Eid et al.	2019	SR	IR	NR	SR	SR	SR	NR	SR	SR
Eid et al.	2019	SR	NR	NR	SR	SR	SR	NR	SR	SR
Eid et al.	2019	SR	IR	NR	SR	SR	SR	NR	SR	SR
Pang et al.	2019	SR	IR	NR	SR	SR	SR	NR	SR	SR
da Costa et al.	2017	SR	IR	NR	SR	SR	SR	NR	SR	SR
Passos et al.	2017	SR	IR	NR	SR	SR	SR	NR	SR	SR
Tsintsadze et al.	2017	SR	IR	NR	SR	SR	SR	NR	SR	SR

*IR* insufficiently reported, *NR* not reported, *SR* sufficiently reported.

## Discussion

The four primary outcomes measured in this study were bond strength, catastrophic failure, fracture resistance and cement film thickness of CAD-CAM fibre posts compared to other types of posts. Bond strength is defined as the force that is required to dislodge or detach posts from root canal dentin. It can be measured by employing a push-out, pull-out, or micro tensile tests [56, 57]. However, results from recent studies have indicated that push-out tests reveal more reliable results when compared to other tests [58, 59]. Bond strength is influenced by various chemical and mechanical factors related to the dentin, post-material, post-treatment and the cement used [60–71]. CAD-CAM posts exhibited higher bond strengths compared to pre-fabricated posts. This result may be attributed to the precision fit of the CAD-CAM posts as these are customized to closely mimick the root canal configuaration in comparison to a pre-fabricated post. The close adapatation of the post morphology to the root canal space further ensures a uniform cement space [63, 72, 73]. Compatible bonding and elevated surface energy may result in better bonding for CAD-CAM and cast metal posts respectively [74, 75]. Primer coating of the post surface has been shown to enhance the surface roughness of CAD-CAM posts, whereas sandblasting can be used to the same effect in cast metal posts [63, 72]. On the contrary, pre-fabricated posts primarily depend on micromechanical retention, leading to inferior bonding [76]. Based on the overall comparison, CAD-CAM posts was ranked the highest for bond strength per the SUCRA ranking. This may be attributed to a robust manufacturing process with enhanced quality control and highly compatible bonding properties, allowing for chemical and micromechanical bonding [74].

Catastrophic failure indicates irreparable damage to the tooth restoration complex [72]. CAD-CAM posts demonstrated a lower risk of catastrophic failure than pre-fabricated posts, whereas cast metal posts demonstrated a higher risk of failure than CAD-CAM posts as per pairwise results. This may be attributed to the unequal distribution of masticatory forces in pre-fabricated posts due to the non-uniformity of the canal morphology, which leads to variable cement thickness and stress concentration [63, 67, 73]. CAD-CAM posts is custom-made to the canal configuration, resulting in a superior fit [72] and uniform cement thickness [74]. The material incompatibility concerning the similarity in the elastic modulus is worst for a cast metal post, which puts the cast metal post at high risk for catastrophic failure due to variable stress concentration [77, 78]. Further a cast metal post requires a significantly higher amount of tooth removal, resulting in weakened tooth structure [73]. Inaccuracies may also result from the casting procedure for cast metal posts [73]. CAD-CAM posts have previously demonstrated superior fatigue resistance when subjected to cyclic loading, leading to the formation of cracks and, consequently, failure [75, 77]. A higher rate of manufacturing errors is attributed to cast metal posts compared to CAD-CAM posts [63, 72].

Cement film thickness refers to the layer of adhesive cement thickness present between the dentinal wall of the root canal and the post. It can influence post adhesion by aiding in retention and fit of the post [67, 70], presence of voids, mechanical stability of stress distribution and fracture resistance [66, 79] and bond strength depending on the amount of cement film present [73, 80]. However, based on the results from our network meta-analysis, the unreliable heterogeneity proves that cement film thickness has no significant effect when comparing CAD to other post materials.

Fracture resistance indicates the ability of the post to resist fracture or withstand forces without breaking when used to restore a tooth that was previously root canal treated. Fracture resistance is measured by fracture or fatigue testing to assess the stiffness and durability of the post system in the root canal [27, 41, 43, 50, 51, 54, 55] by placing the post under different conditions or environments. Based on the meta-analysis results, fracture resistance of CAD was comparable to PFP and CMP. Material properties alone might not play a significant role in the fracture resistance of posts. Another external factor that could affect the fracture resistance of post systems in the root canals is the type of cement used. Adhesive resin cement has proven to bond better and provide support for posts to dentinal walls [80, 81]. The remaining dentin thickness supports the post. The presence of lesser remaining dentine thickness leads to a higher risk of post-fracture [82]. Accurate post-space preparation for post-fitting is an essential factor in minimising the risk of fracture of the post or tooth structure due to proper force distribution from the post towards the tooth [60, 70]. The tooth anatomy and location contribute to stress distribution from masticatory forces and functional loading [83–85].

The current network meta-analysis’s limitations include the inclusion of in vitro studies. However, it provides an up-to-date comprehensive comparison of the different post-types.

Further high-quality in vitro studies, including recently introduced CAD-CAM post materials, are recommended to evaluate the comparative mechanical properties of different post-types. Clinicians need to carefully evaluate the material properties of the CAD-CAM posts before making appropriate comparisons in long-term clinical studies. Subsequently, prospective cohort clinical studies may provide the most reliable evidence of different post-types survival and clinical performance.

## Conclusion

CAD-CAM fibre posts demonstrate higher bond strength, lower catastrophic failure rates and similar fracture resistance compared to pre-fabricated fibre posts and cast metal posts in in vitro studies. Results from the studies need to be validated with further high-quality in vitro studies and randomised controlled clinical trials.

## Supplementary information

Supplementary Information

PRISMA Checklist

## Data Availability

The authors confirm that the data supporting the findings of this study are available within the article [and/or] its supplementary materials.

## References

[1] Al-Omiri MK, Mahmoud AA, Rayyan MR, Abu-Hammad O. Fracture resistance of teeth restored with post-retained restorations: an overview. J Endod. 2010;36:1439–49. 20728706 10.1016/j.joen.2010.06.005

[2] Dangra Z, Gandhewar M. All about Dowels - A Review Part I. Considerations before cementation. J Clin Diagn Res. 2017;11:ZG06–ZG11. 28969296 10.7860/JCDR/2017/26472.10518 PMC5620937

[3] Morgano SM, Milot P. Clinical success of cast metal posts and cores. J Prosthet Dent. 1993;70:11–6. 8366452 10.1016/0022-3913(93)90030-r

[4] Theodosopoulou JN, Chochlidakis KM. A systematic review of dowel (post) and core materials and systems. J Prosthodont. 2009;18:464–72. 19500237 10.1111/j.1532-849X.2009.00472.x

[5] Heydecke G, Butz F, Strub JR. Fracture strength and survival rate of endodontically treated maxillary incisors with approximal cavities after restoration with different post and core systems: an in-vitro study. J Dent. 2001;29:427–33. 11520592 10.1016/s0300-5712(01)00038-0

[6] Mekayarajjananonth T, Kiat-amnuay S, Salinas TJ. A combined direct dowel and indirect core technique. Quintessence Int. 2000;31:19–23. 11203901

[7] Schwartz RS, Robbins JW. Post placement and restoration of endodontically treated teeth: a literature review. J Endod. 2004;30:289–301. 15107639 10.1097/00004770-200405000-00001

[8] Awad MA, Marghalani TY. Fabrication of a custom-made ceramic post and core using CAD-CAM technology. J Prosthet Dent. 2007;98:161–2. 17692598 10.1016/S0022-3913(07)60050-X

[9] Tortopidis D, Kourtis S, Kountouras K. Restoration of endodontically treated anterior teeth with cast metallic post or pre-fabricated fibre post placement: 2 case reports and critical literature review. Balk J Dent Med. 2015;19:86–91.

[10] Baba NZ, Goodacre CJ, Daher T. Restoration of endodontically treated teeth: the seven keys to success. Gen Dent. 2009;57:596–603. 19906612

[11] Hayashi M, Takahashi Y, Imazato S, Ebisu S. Fracture resistance of pulpless teeth restored with post-cores and crowns. Dent Mater. 2006;22:477–85. 16171857 10.1016/j.dental.2005.03.017

[12] Mezzomo E, Massa F, Libera SD. Fracture resistance of teeth restored with two different post-and-core designs cemented with two different types of cement: an in-vitro study. Part I. Quintessence Int. 2003;34:301–6. 12731618

[13] Baba NZ, Goodacre CJ Treatment Options and Materials for Endodontically Treated Teeth. In Contemporary Restoration of Endodontically Treated Teeth: Evidence-Based Diagnosis and Treatment Planning. 1st ed., Hanover Park, IL: Quintessence Publishing. 2013.

[14] Monaghan P, Roh L, Kim J. Corrosion behaviour of selected implant alloys (abstract 1177). J Dent Res. 1992;71:253.

[15] Hulbert SF, Morrison SJ, Klawitter JJ. Tissue reaction to three ceramics of porous and non-porous structures. J Biomed Mater Res. 1972;6:347–74. 4116127 10.1002/jbm.820060505

[16] Porter DL, Heuer AH. Mechanism of toughening partially stabilised zirconia ceramics (PSZ). J Am Ceram Soc Discuss Notes. 1977;60:183–4.

[17] Ichikawa Y, Akagawa Y, Nikai H, Tsuru H. Tissue compatibility and stability of a new zirconia ceramic in vivo. J Prosthet Dent. 1992;68:322–6. 1501183 10.1016/0022-3913(92)90338-b

[18] Dietschi D, Romelli M, Goretti A. Adaptation of adhesive posts and cores to dentin after fatigue testing. Int J Prosthodont. 1997;10:498–507. 9495169

[19] Hedlund SO, Johansson NG, Sjögren G. Retention of pre-fabricated and individually cast root canal posts in-vitro. Br Dent J. 2003;195:155–8. discussion 147. 12907984 10.1038/sj.bdj.4810405

[20] Duret B, Reynaud M, Duret F. Un nouveau concept de reconstitution corono-radiculaire: le Composipost (1) [New concept of coronoradicular reconstruction: the Composipost (1)]. Chir Dent Fr. 1990;60:131–41. 2272223

[21] Lamichhane A, Xu C, Zhang FQ. Dental fibre-post resin base material: a review. J Adv Prosthodont. 2014;6:60–5. 24605208 10.4047/jap.2014.6.1.60 PMC3942529

[22] Jawed A, Alghmlas AS, Khurshid Z Fibre post: Physics, chemistry, adhesive properties, and implications for root canal retreatment. In: Khurshid Z, ed. Fibre Reinforced Composites in Dental Applications. Elsevier; 2022:357-78.

[23] Plotino G, Grande NM, Bedini R, Pameijer CH, Somma F. Flexural properties of endodontic posts and human root dentin. Dent Mater. 2007;23:1129–35. 17116326 10.1016/j.dental.2006.06.047

[24] Bru E, Forner L, Llena C, Almenar A. Fibre post behaviour prediction factors. A review of the literature. J Clin Exp Dent. 2013;5:e150–3. 24455071 10.4317/jced.50619 PMC3892248

[25] Cormier CJ, Burns DR, Moon P. In-vitro comparison of the fracture resistance and failure mode of fibre, ceramic, and conventional post systems at various stages of restoration. J Prosthodont. 2001;10:26–36. 10.1111/j.1532-849x.2001.00026.x. 11406793 10.1111/j.1532-849x.2001.00026.x

[26] Giok KC, Veettil SK, Menon RK. Comparative effectiveness of fiber and metal posts in the restoration of endodontically treated teeth: A systematic review with network meta-analysis. J Prosthet Dent. 2023;S0022-3913:00569-3. 10.1016/j.prosdent.2023.08.022. 10.1016/j.prosdent.2023.08.022 37827970

[27] da Costa RG, Freire A, Caregnatto de Morais EC, Machado de Souza E, Correr GM, Rached RN. Effect of CAD/CAM glass fibre post-core on cement micromorphology and fracture resistance of endodontically treated roots. Am J Dent. 2017;30:3–8. 29178707

[28] Costa TS, Brandão RMR, Farias VBC, SoutoMaior JR A systematic review of CAD-CAM glass fibre compared with conventional pre-fabricated glass fibre posts. J Prosthet Dent. 2022:S0022-391300053-1. 10.1016/j.prosdent.2022.01.007 35933174

[29] Gutiérrez MA, Guerrero CA, Baldion PA. Efficacy of CAD/CAM glass fibre posts for restoration endodontically treated teeth. Int J Biomater. 2022;2022:8621835. 35096068 10.1155/2022/8621835 PMC8799365

[30] Al-Qarni FD. Customised post and cores fabricated with CAD/CAM technology: a literature review. Int J Gen Med. 2022;15:4771–9. 35571288 10.2147/IJGM.S365296 PMC9091696

[31] Mishra SK, Chowdhary R. Current Evidence on the CAD-CAM-fabricated glass fibre post. Int J Prosthodont Restor Dent. 2022;12:103–103.

[32] Page MJ, Moher D, Bossuyt PM, Boutron I, Hoffmann TC, Mulrow CD, et al. PRISMA 2020 explanation and elaboration: updated guidance and exemplars for reporting systematic reviews. BMJ. 2021;372:n160 10.1136/bmj.n160. 33781993 10.1136/bmj.n160 PMC8005925

[33] Delgado AH, Sauro S, Lima AF, Loguercio AD, Della Bona A, Mazzoni A, et al. RoBDEMAT: A risk of bias tool and guideline to support reporting of pre-clinical dental materials research and assessment of systematic reviews. J Dent. 2022;127:104350. 36341980 10.1016/j.jdent.2022.104350

[34] Veroniki AA, Jackson D, Viechtbauer W, Bender R, Bowden J, Knapp G, et al. Methods to estimate the between-study variance and its uncertainty in meta-analysis. Res Synth Methods. 2016;7:55–79. 26332144 10.1002/jrsm.1164 PMC4950030

[35] Petropoulou M, Mavridis D. A comparison of 20 heterogeneity variance estimators in statistical synthesis of results from studies: a simulation study. Stat Med. 2017;36:4266–80. 28815652 10.1002/sim.7431

[36] Caldwell DM, Ades AE, Higgins JP. Simultaneous comparison of multiple treatments: combining direct and indirect evidence. BMJ. 2005;331:897–900. 16223826 10.1136/bmj.331.7521.897 PMC1255806

[37] Hoaglin DC, Hawkins N, Jansen JP, Scott DA, Itzler R, Cappelleri JC, et al. Conducting indirect-treatment-comparison and network-meta-analysis studies: report of the ISPOR Task Force on Indirect Treatment Comparisons Good Research Practices: part 2. Value Health. 2011;14:429–37. 21669367 10.1016/j.jval.2011.01.011

[38] Raudenbush SW Analyzing effect sizes: Random-effects models. In: Cooper H, Hedges LV, Valentine JC, editors. The handbook of research synthesis and meta-analysis. 2nd ed. New York: Russell Sage Foundation; 2009. p. 295-315.

[39] Chaimani A, Higgins JP, Mavridis D, Spyridonos P, Salanti G. Graphical tools for network meta-analysis in STATA. PLoS One. 2013;8:e76654. 10.1371/journal.pone.0076654. 10.1371/journal.pone.0076654 PMC3789683 24098547

[40] Watt J, Tricco AC, Straus S, Veroniki AA, Naglie G, Drucker AM. Research techniques made simple: network meta-analysis. J Investig Dermatol. 2019;139:4–12.e1. 30579427 10.1016/j.jid.2018.10.028

[41] Pang J, Feng C, Zhu X, Liu B, Deng T, Gao Y, et al. Fracture behaviors of maxillary central incisors with flared root canals restored with CAD/CAM integrated glass fibre post-and-core. Dent Mater J. 2019;38:114–9. 30381631 10.4012/dmj.2017-394

[42] Tsintsadze N, Juloski J, Carrabba M, Tricarico M, Goracci C, Vichi A, et al. Performance of CAD/CAM fabricated fibre posts in oval-shaped root canals: an in-vitro study. Am J Dent. 2017;30:248–54. 29178727

[43] Eid R, Juloski J, Ounsi H, Silwaidi M, Ferrari M, Salameh Z. Fracture resistance and failure pattern of endodontically treated teeth restored with computer-aided design/computer-aided manufacturing post and cores: a pilot study. J Contemp Dent Pr. 2019;20:56–63. 31102396

[44] Passos L, Barino B, Laxe L, Street A. Fracture resistance of single-rooted pulpless teeth using hybrid CAD/CAM blocks for post and core restoration. Int J Comput Dent. 2017;20:287–301. 28852746

[45] Eid R, Azzam K, Ounsi H. Influence of adaptation and adhesion on the retention of computer-aided design/computer-aided manufacturing glass fibre posts to root canal. J Contemp Dent Pr. 2019;1:1003–8. 31797819

[46] Eid RY, Koken S, Baba NZ, Ounsi H, Ferrari M, Salameh Z. Effect of fabrication technique and thermal cycling on the bond strength of CAD/CAM milled custom fit anatomical post and cores. -Vitr study J Prosthodont 2019;28:898–905. 10.1111/jopr.13101 31397947

[47] Saisho H, Marcolina G, Perucelli F, Goulart da Costa R, Machado de Souza E, Nunes RR. Fracture strength, pull-out bond strength, and volume of luting agent of tooth-colored CAD-CAM post-and-cores. J Prosthet Dent. 2023;129:599–606. 36127164 10.1016/j.prosdent.2022.06.012

[48] Nodehi D, Moraditalab A, Shafiee S, Sekandari S, Ahrari F. Fracture resistance of endodontically treated premolars reconstructed by traditional casting and CAD-CAM milling post and cores. Int J Dent. 2022;2022:6736303. 36249731 10.1155/2022/6736303 PMC9553496

[49] Ming X, Zhang Z, Xie W, Zhang Y, Li Y, Zhang W. Internal adaptation and mechanical properties of CAD/CAM glass fibre post-cores in molars: An in-vitro study. J Dent. 2023;138:104685. 37659715 10.1016/j.jdent.2023.104685

[50] Ghanem N, Baherly N, Hassan H. Fracture resistance of custom-milled CAD/CAM post and core using two different materials: an in-vitro study. J Stomatol. 2022;75:207–15.

[51] Taques LV, Chidoski JC, Ávila BO, Jitumori RT, Gomes JC, Gomes GM, et al. Evaluation of bond strength and fracture load of teeth with flared root canals restored with pre-fabricated, anatomical, and computer-aided design and computer-aided manufacturing fiber posts. Oper Dent. 2023;48:524–37. 37635454 10.2341/22-083-L

[52] Pinto APS, França FMG, Basting RT, Turssi CP, Rodrigues Júnior JJ, Amaral FLB. Effect of endodontic sealers on push-out bond strength of CAD-CAM or prefabricated fiber glass posts. Braz Oral Res. 2023;37:e052. 10.1590/1807-3107bor-2023.vol37.0052. 10.1590/1807-3107bor-2023.vol37.0052 37255072

[53] Abdelkader AM. Effect of CAD-CAM and pre-fabricated glass fiber post adaptation on the push-out bond strength to root canal dentin. Egypt Dent J. 2022;68:2487–95.

[54] Gama MS, Portela L, Patinha C, Durães N. CAD/CAM milled glass fiber posts: Adaptation and mechanical behavior in flared root canals. Oper Dent. 2021;46:438–47. 34624118 10.2341/20-198-L

[55] Seckler IN, da Silveira Bueno CE, Kato AS, Pinheiro SL, Lima DANL, de Souza, DFS et al. Impact of mechanical load of three post and core systems: CAD/CAM-fabricated glass fiber, pre-fabricated glass fiber customized with composite resin, and cast metal posts and cores. Cons Dent Endod J. 2020;5:36–41.

[56] Pereira JR, Pamato S, Santini MF, Porto VC, Ricci WA, Só MVR. Push-out bond strength of fibreglass posts cemented with adhesive and self-adhesive resin cements according to the root canal surface. Saudi Dent J. 2021;33:22–26. 33473238 10.1016/j.sdentj.2019.11.009 PMC7801235

[57] Habib SR, Ansari AS, Khan AS, Alamro NM, Alzaaqi MA, Alkhunefer YA, AlHelal AA, Alnassar TM, Alqahtani AS. Push-out bond strength of endodontic posts cemented to extracted teeth: an in-vitro evaluation. Mater (Basel). 2022;15:6792. 10.3390/ma15196792 PMC9572078 36234146

[58] Altmann AS, Leitune VC, Collares FM. Influence of eugenol-based sealers on push-out bond strength of fibre post luted with resin cement: systematic review and meta-analysis. J Endod. 2015;41:1418–23. 26211566 10.1016/j.joen.2015.05.014

[59] Mastoras K, Vasiliadis L, Koulaouzidou E, Gogos C. Evaluation of push-out bond strength of two endodontic post systems. J Endod 2012;38:510–4. 22414839 10.1016/j.joen.2011.12.039

[60] Pereira JR, Rosa RA, Soares RG, Azevedo FA, Vansan LP. Effect of post space preparation on bond strength of fibre posts to root dentin. J Endod. 2011;37:1285–7.

[61] Ferrari M, Vichi A. Retrospective study of the clinical performance of fibre posts. Am J Dent. 2002;15:311–5. 11763869

[62] Bitter K, Paris S, Putz K. Effects of silanization on bond strengths of fibre posts. Dent Mater. 2006;22:752–8. 16427122 10.1016/j.dental.2005.11.002

[63] Goracci C, Ferrari M. Current perspectives on post systems: a literature review. Aust Dent J. 2011;56:77–83. 21564118 10.1111/j.1834-7819.2010.01298.x

[64] Monticelli F, Grandini S, Goracci C, Ferrari M. Clinical behavior of translucent-fibre posts: a 2-year prospective study. Int J Prosthodont. 2003;16:593–6. 14714836

[65] Ferracane JL. Resin composite-state of the art. Dent Mater. 2011;27:29–38. 21093034 10.1016/j.dental.2010.10.020

[66] Radovic I, Monticelli F, Goracci C, Vulicevic ZR, Ferrari M. Self-adhesive resin cements: a literature review. J Adhes Dent. 2008;10:251–8. 18792695

[67] Grandini S, Goracci C, Monticelli F, Ferrari M. Clinical evaluation of the use of fibre posts and direct resin restorations for endodontically treated teeth. Int J Prosthodont. 2005;18:399–404. 16220805

[68] Mazzitelli C, Monticelli F, Toledano M, Ferrari M, Osorio R. Dentin treatment effects on the bonding performance of self-adhesive resin cements. Eur J Oral Sci. 2008;116:253–9. 10.1111/j.1600-0722.2009.00703.x 20156269

[69] Perdigão J, Gomes G, Lopes M. The effect of silane on the bond strengths of fibre posts. Dent Mater. 2006;22:752–8. 16427122 10.1016/j.dental.2005.11.002

[70] Faria-e-Silva AL, de Menezes MS, Silva-Neto A, Reis GR. Influence of cement type and relining procedure on push-out bond strength of fibre posts after cyclic loading. J Adhes Dent. 2014;16:87–92. 24027772 10.3290/j.jad.a30556

[71] Zicari F, Van Meerbeek B, Scotti R, Naert I. Effect of thermal cycling and load cycling on the bond strength of a fibre post bonded with a self-adhesive resin cement. Clin Oral Investig. 2013;17:717–24.

[72] Ferrari M, Vichi A, Garcia-Godoy F. Clinical evaluation of fibre-reinforced epoxy resin posts and cast post and cores. Am J Dent. 2000;13:15B–18B. 11763866

[73] Pereira JR, et al. Effect of post space preparation on the retention of pre-fabricated posts. J Oral Rehabil. 2006;33:142–6.

[74] Valandro LF, Yoshiga S, de Melo RM, Galhano GA, Mallmann A, Marinho CP, et al. Microtensile bond strength between a quartz fibre post and a resin cement: Effect of post surface conditioning. J Adhes Dent. 2006;8:105–11. 16708722

[75] Grandini S, Goracci C, Monticelli F, Tay FR, Ferrari M. Fatigue resistance and structural characteristics of fibre posts: Three-point bending test and SEM evaluation. Dent Mater. 2005;21:75–82. 15681005 10.1016/j.dental.2004.02.012

[76] Tay FR, Pashley DH. Monoblocks in root canals: a hypothetical or tangible goal. J Endod 2007;33:391–8. 17368325 10.1016/j.joen.2006.10.009 PMC2223075

[77] Schmitter M, Rammelsberg P, Gabbert O, Ohlmann B. Influence of clinical baseline findings on the survival of 2 post systems: a randomized clinical trial. Int J Prosthodont. 2007;20:173–8. 17455439

[78] Bittner N, Hill T, Randi A. Comparison of the failure modes of fibre posts and custom-made posts. Endod Dent Traumatol. 2002;18:1–7.

[79] Monticelli F, Goracci C, Grandini S, Ferrari M. Scanning electron microscopic evaluation of fibre post-resin root canal dentin interfaces. J Endod. 2003;29:871–5.

[80] Goracci C, Tavares AU, Fabianelli A, Monticelli F, Raffaelli O, Cardoso PC, Ferrari M. The adhesion between fibre posts and root canal walls: comparison between microtensile and push-out bond strength measurements. Eur J Oral Sci. 2004;112:353–61. 15279655 10.1111/j.1600-0722.2004.00146.x

[81] Sahafi A, Peutzfeldt A, Asmussen E, Gotfredsen K. Retention and failure morphology of pre-fabricated posts. Int J Prosthodont. 2004;17:307–12. 15237877

[82] Ferrari M, Cagidiaco MC, Goracci C, Vichi A, Mason PN, Radovic I, Tay F. Long-term retrospective study of the clinical performance of fibre posts. Am J Dent. 2007;20:287–91. 17993023

[83] Akkayan B, Gulmez T. Resistance to fracture of endodontically treated teeth restored with different post systems. J Prosthet Dent. 2002;87:431–7. 12011860 10.1067/mpr.2002.123227

[84] Torbjörner A, Karlsson S, Ödman PA. Survival rate and failure characteristics for two post designs. J Prosthet Dent. 1995;73:439–44. 7658393 10.1016/s0022-3913(05)80072-1

[85] Mannocci F, Cowie J. Restoration of endodontically treated teeth. Br Dent J. 2014;216:341–6. 24651340 10.1038/sj.bdj.2014.198

