# Supplementary material for: Do CAD-CAM fibre posts exhibit higher bond strength and fracture resistance than other types of posts? systematic review with network meta-analysis of in-vitro studies
Source: BDJ Open. 2025 Mar 17;11:25. doi: 10.1038/s41405-025-00315-x (PMC11914252; doi:10.1038/s41405-025-00315-x)
Supplement: Supplementary file 1 — Supplementary Information [file 41405_2025_315_MOESM1_ESM.pdf]

Supplementary Information

Supplementary Figure 1. League table depicting the combined pairwise and network meta-analysis results for bond strength

**CAD**	**-9.29 (6.95, -25.53)**	**-2.63 (-0.82, -4.44)**
**-0.55 (-3.15,2.05)**	**CMP**	**-18.22 (-3.92, -33.52) ***
**1.10 (0.02,2.18)**	**1.65 (-0.96,4.26)**	**PFP**

Abbreviations: CAD-CAD-CAM fibre posts, PFP-Pre-fabricated fibre posts, CMP-Cast metal posts

Supplementary Figure 2. Funnel plot depicting publication bias for bond strength

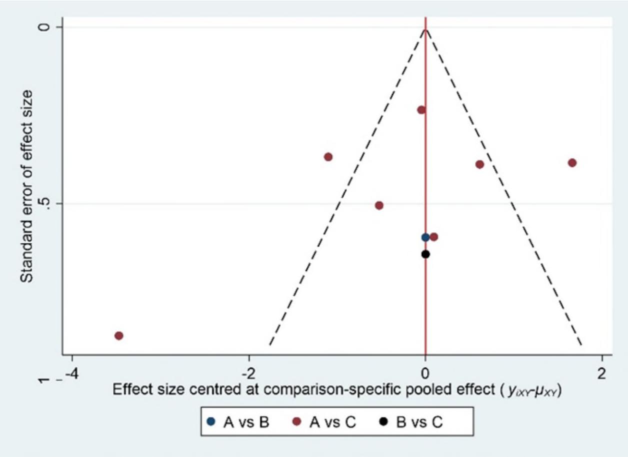

Supplementary Figure 3. League table depicting the combined pairwise and network meta-analysis results for catastrophic failures

AMA	0.67 (0.18, 2.42)	1.33 (0.61, 2.91)	
1.87 (0.74,4.73)	CAD	2.29 (1.13, 4.62)	-0.66 (-0.39, - 1.12)
0.74 (0.34,1.60)	0.39 (0.23,0.69)	CMP	-1.88 (-0.93, - 3.79)
1.24 (0.49,3.19)	0.67 (0.41,1.08)	1.69 (0.97,2.95)	PFP

Abbreviations: CAD-CAD-CAM fibre posts, PFP-Pre-fabricated fibre posts, CMP-Cast metal posts, Amalgam cores -AMA

Supplementary Figure 4. Funnel plot depicting publication bias for catastrophic failures

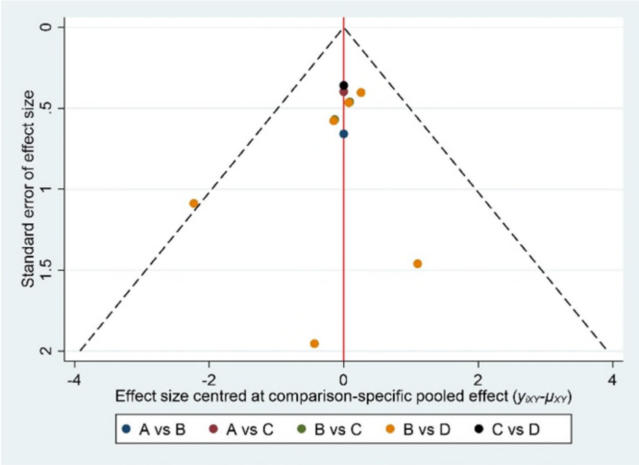

Supplementary Figure 5. Forest plot depicting the pairwise meta-analysis results for fracture resistance

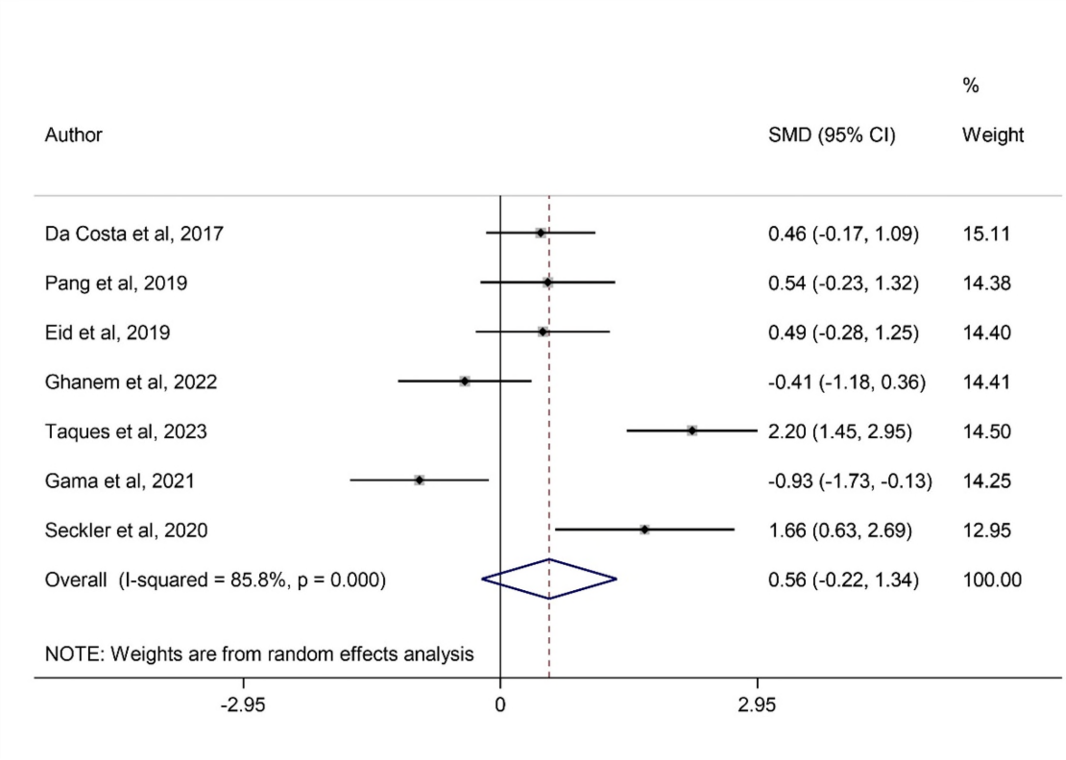

Supplementary Figure 6. Network plot depicting the network meta-analysis results for cement film thickness

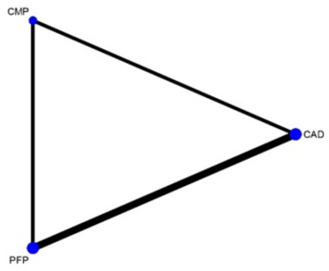

Abbreviations: CAD-CAD-CAM fibre posts, PFP-Pre-fabricated fibre posts, CMP-Cast metal posts

Supplementary Figure 7. SUCRA ranking curves for cement film thickness

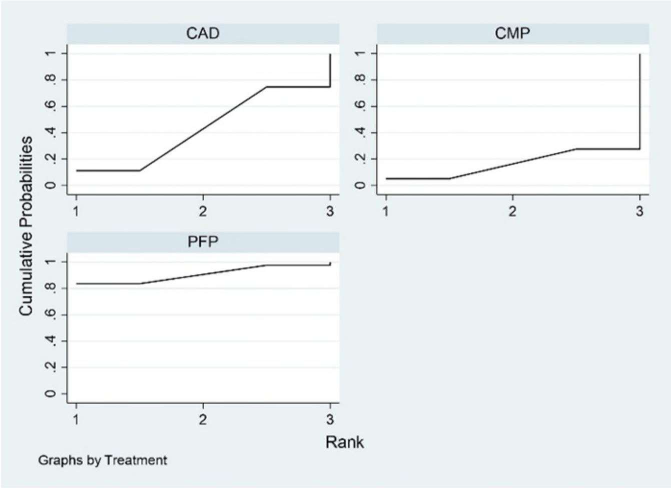

Abbreviations: CAD-CAD-CAM fibre posts, PFP-Pre-fabricated fibre posts, CMP-Cast metal posts

Supplementary Figure 8. Forest plot depicting the pairwise meta-analysis results for cement film thickness

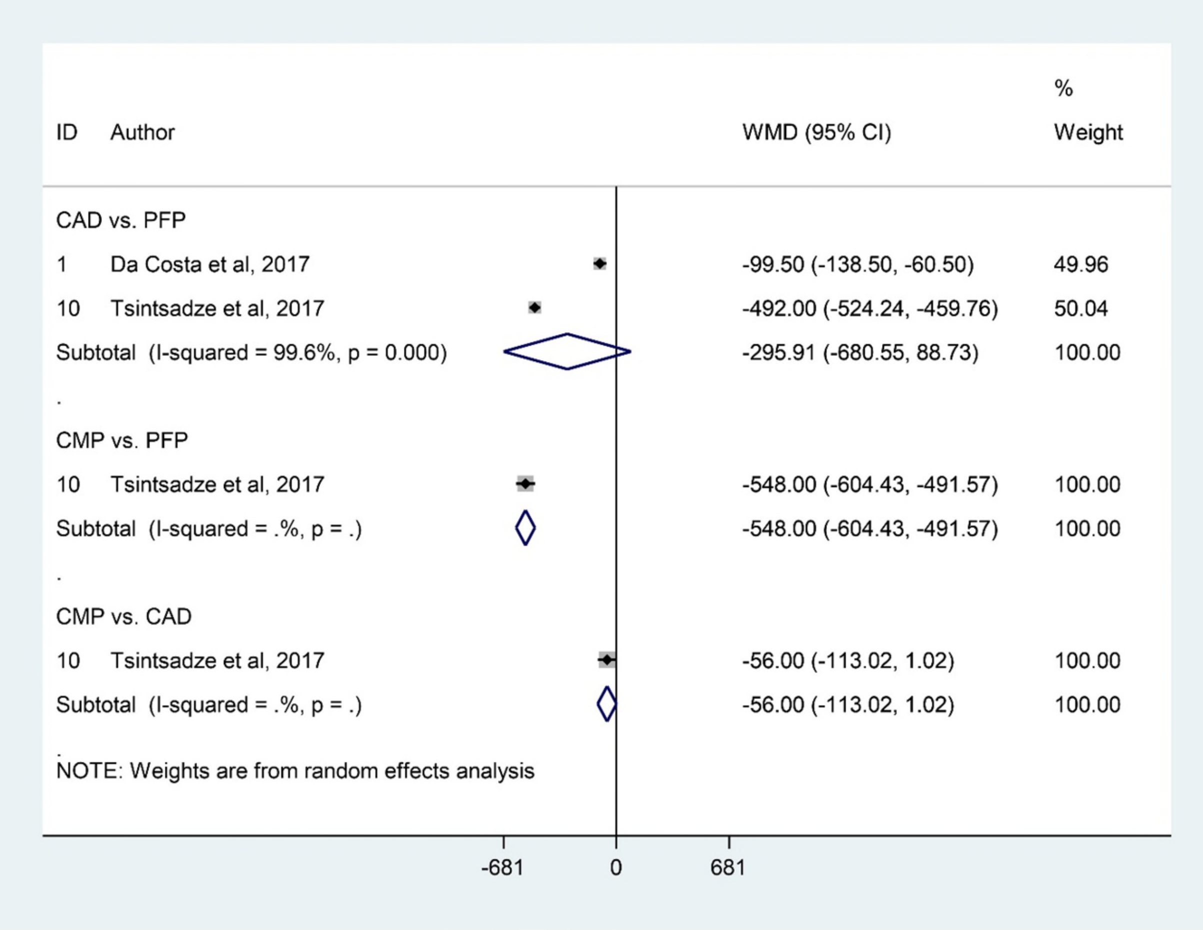

Abbreviations: CAD-CAD-CAM fibre posts, PFP-Pre-fabricated fibre posts, CMP-Cast metal posts

Supplementary Table 1. Network meta-analysis results and ranking for bond strength

Interventions	Hardness		
	SMD (95% CI)	P	SUCRA rank (score)
**CAD**	1.09 (0.01, 2.17)	0.046	2 (29.8)
**PFP**	Reference		3 (0.4)
**CMP**	1.65 (-0.95, 4.25)	0.21	1 (69.8)

Abbreviations: CAD-CAD-CAM fibre posts, PFP-Pre-fabricated fibre posts, CMP-Cast metal posts

Supplementary Table 2. Network meta-analysis results and ranking for catastrophic failures

Interventions	Hardness		
	SMD (95% CI)	P	SUCRA rank (score)
**CAD**	0.66 (0.40, 1.08)	0.10	1 (86.0)
**PFP**	Reference		3 (3.4)
**CMP**	1.68 (0.96, 2.95)	0.06	4 (0)
**AMA**	1.24 (0.48, 3.18)	0.64	2 (10.6)

Abbreviations: CAD-CAD-CAM fibre posts, PFP-Pre-fabricated fibre posts, CMP-Cast metal posts, Amalgam cores-AMA
